# Compatible solute influence on nucleic acids: Many questions but few answers

**DOI:** 10.1186/1746-1448-4-6

**Published:** 2008-06-03

**Authors:** Matthias Kurz

**Affiliations:** 1 Institut für Mikrobiologie & Biotechnologie, Rheinische Friedrich Wilhelms-Universität Bonn, Meckenheimer Allee 168, 53115 Bonn, Germany

## Abstract

Compatible solutes are small organic osmolytes including but not limited to sugars, polyols, amino acids, and their derivatives. They are compatible with cell metabolism even at molar concentrations. A variety of organisms synthesize or take up compatible solutes for adaptation to extreme environments. In addition to their protective action on whole cells, compatible solutes display significant effects on biomolecules *in vitro*. These include stabilization of native protein and nucleic acid structures. They are used as additives in polymerase chain reactions to increase product yield and specificity, but also in other nucleic acid and protein applications.

Interactions of compatible solutes with nucleic acids and protein-nucleic acid complexes are much less understood than the corresponding interactions of compatible solutes with proteins. Although we may begin to understand solute/nucleic acid interactions there are only few answers to the many questions we have. I summarize here the current state of knowledge and discuss possible molecular mechanisms and thermodynamics.

## Background

**Figure 1:**
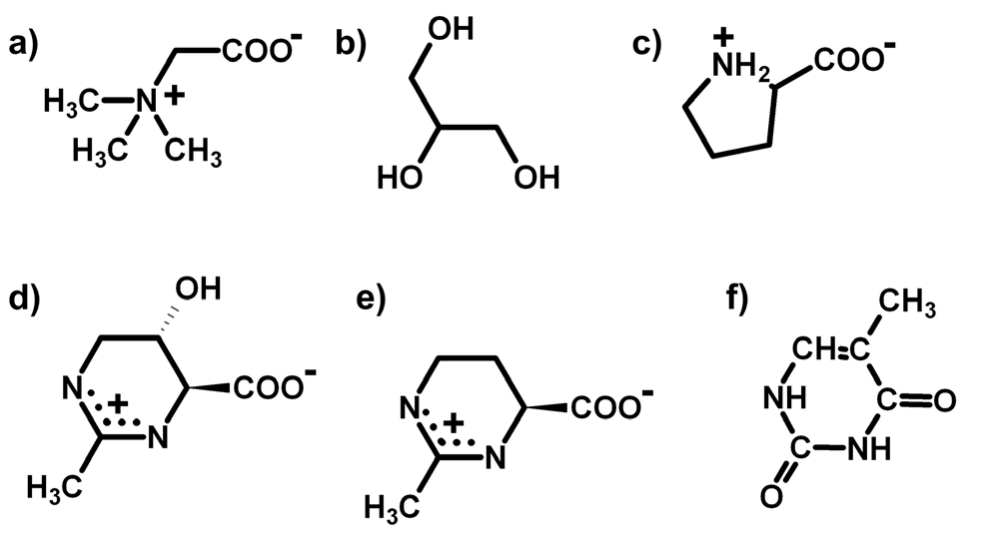
**Sample compatible solutes**. a) Glycine betaine (usually abbreviated as *betaine*), b) glycerol, c) proline d) hydroxyetoine (also designated THP A) and e) ectoine (also designated THP B). The THPs (for tetrahydropyrimidine) have a structural similarity to the pyrimidine bases, see f) thymine as example. Note that the aromatic thymine ring is planar whereas the cyclic THPs are in half-chair conormation.

Compatible solutes (CS) are small organic osmolytes including sugars, polyols, amino acids and their derivatives. They are compatible with cellular metabolism even at molar concentrations. (See figure 1 for a few examples). As reviewed extensively elsewhere [1-3], CS are found in microorganisms from all three domains: A*rchaea*, B*acteria *and E*ucarya*, but also in higher organisms and are used in a wide range of applications [4]. A complete list of disciplines interested in compatible solutes would start with halophilic/osmophilic bacteria [5,6] and yeasts [7], their bioenergetics [8] and their relevance for bio-remediation [9]. But the list would further extend to medical disciplines dealing with for example cancer research [10] or dermatology [11,12]. Even food science takes an interest in CS research, very recent findings demonstrate that CS can be found as a natural component of food traditionally processed by microorganisms [13]. Therefore it is not surprising, that research on solute effects on macromolecules is widely spread. Most of it has been performed in the field of proteins. Beneficiary effects of compatible solutes on proteins *in vitro *have been extensively studied (e.g. [14-17]) as have been effects on protein expression [18] and stabilization of whole cells [19,20]. Research on protein stability and protein stabilization by compatible solutes has led to the development of some theories (and variations thereof) concerning solute/protein interactions. The four most outstanding among them discuss preferential interaction [21], water replacement [22], water density fractions [23] and osmophobic effects [24] as the mechanisms of solute/protein interactions. However, this short review can *not *serve as a comprehensive review of these theories and their applications. Therefore I will present and discuss only the underlying ideas and their application to nucleic acids. More attention will be given to recent data relevant for solute/nucleic acid interactions [25] and on the background of these findings.

Beneficial effects of compatible solutes on nucleic acids and nucleic acid/protein complexes are mainly known from improvements in yield and specificity of polymerase chain reaction (PCR), see [26-32] for examples. But effects also extend to nucleic acid stabilization [33], improvement of protein/nucleic acid complex formation [34], nucleic acid purification [35] and cell free transcription [36,37] as well as modulation of restriction enzyme function [38,39]. Contrary to other well known effector molecules like polyamines which stabilize negatively charged macromolecules due to their cationic nature [40], the mode of interaction of zwitterionic, anionic and uncharged low molecular weight compounds with nucleic acids is barely understood. There are some obvious possibilities how they might influence nucleic acids. Among them are changes in the electrostatic environment [41], intercalation [42] and a role as anti-intercalators [43].

In this review I will start with an overview of known effects of compatible solutes on nucleic acids, focusing on double stranded DNA since a wealth of data can be retrieved from this area of work. Still "DNA comes in many forms" [44], a fact we are aware of since 1957 [45], only four years after Watson and Crick presented their theory of the double stranded DNA helix [46]. Therefore I am also going to present more complex structures like triple- and quadruple helices. Considering the importance of riboswitches [47] and the recent advances which have been made in this field [48] interference of small metabolites with RNA has become of prime importance. Therefore RNA interactions with small osmolytes, be they direct [49] or indirect, might play an essential role in regulation of compatible solute biosynthesis and adaptation. After discussing potential models for molecular mechanics of interactions, and first steps towards applying those models to nucleic acids, I will address the questions still unanswered. A few thoughts will be given towards computational methods before I draw my conclusions and give some future prospects.

## Inorganic ions in brief

Inorganic ions are not meant to be a major subject of this review. Their influence on nucleic acids, especially RNA, and their structure has been reviewed thoroughly by Draper [50] and newer findings, including CS effects, have been presented recently [25]. Nevertheless, we have to consider effects of inorganic ions when interpreting experimental data since they are an indispensable component of buffers and the native environment of nucleic acids. Most obvious is their influence on DNA stability (see next section), which is reflected in those melting temperature (T_M_) calculations that consider ion concentrations or solvent ionic strength. The approach of Frank-Kamenetskii [51] may serve here as an example:

T_M _(°C) = 87.16 + 0.345 × (%GC) + log [Na+] × (20.17 - 0.066 × (%GC)), [Na+] given in mM, (%GC) is GC content given in a range from 0 to 1.

Therefore cations are of special interest, among them physiologically important species like bivalent magnesium and monovalent sodium and potassium. They act as counterions to the phosphate backbone [52] of nucleic acids and – due to their charge screening effect – reduce repulsive force between the two stands, hence the increase in DNA melting temperature. Cations are able to counteract effects of compatible solutes on nucleic acids and vice versa [25,53]. This phenomenon is also linked to the counterion atmosphere [54,55] and therefore to the counterion condensation theory, originally introduced by Manning [56] and Oosawa [57] and later refined by Shaughnessy [58].

In accordance with their native function in nucleic acid stability and functionality of nucleic acid processing enzymes, Mg^2+ ^and K^+ ^are able to stabilize nucleic acids in *in vitro *assays [36]. But we also already know that, depending on concentrations, bivalent ion species can compact DNA and induce its aggregation [59]. This compaction only occurs in a narrow range of concentrations. Apparently hydration of the cations, especially the size of the hydration shell, plays an important role: Li^+ ^was reported to behave very differently from other monovalent alkali ions [60]. Ions are also known to influence the stability of more exotic DNA structures [61,62] and to be involved in halophilic adaptation of protein DNA interactions [63-65]. This is of importance when comparing and contrasting organisms which accumulate salt for osmoadaptation (*salt-in *strategy) with those using *compatible solutes *(CS strategy). K^+ ^is not only used by *salt-in *organisms (which might have equally high Cl^- ^concentrations inside [66]) but also by compatible solute producers in their early up-shift adaptation phase [67,68]. Organisms using the CS strategy usually employ potassium as a transient solute and glutamate as its counterion. All this shows us that an in vivo model of nucleic acid/compatible solute interactions definitely has to include their ionic environment.

## Thermal DNA melting and DNA stability

A first approach to compatible solute nucleic acid interactions should aim at the effect of compatible solutes on the melting of double stranded DNA (dsDNA).

Many formulas for calculation of dsDNA melting temperature T_M _exist. The Wallace rule [69] is the most simple one, but it is limited to DNA oligomers with 14 to 20 nucleotides and a highly specialized formula which requires the use of certain buffer concentrations. As already mentioned in the previous section, other methods for T_M _calculation include terms for counterion concentrations or ionic strength of the buffer, e.g. [51]. Even more sophisticated models include thermodynamic lattice models and nearest neighbour calculations [70-72].

**Figure 2:**
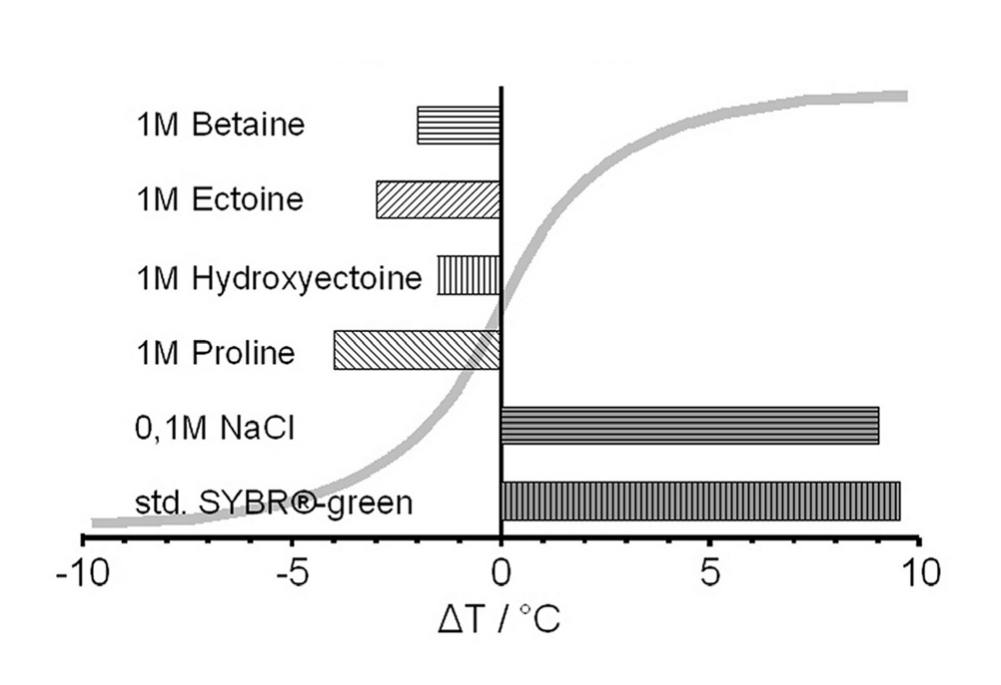
**Solute effects on meting temperature T_M_**. The melting temperature T_M _of double stranded DNA is shifted by solutes. Compatible solutes like betaine, tetrahydropyrimidines and proline lower T_M_. Sodium chloride shows the same shift in opposite direction at concentrations tenfold lower. Dyes interacting with dsDNA like SYBR^®^-green also increase T_M _at those very low concentrations which are used in realtime applications. The applied standard concentrations are not disclosed by the manufacturer. (Kurz and co-workers, unpublished data for 50% GC-content.).

Since 1993 we know that compatible solutes have an effect on thermal melting. Rees and co-workers [53] showed that glycine betaine (or simply betaine) in high concentrations eliminates the GC-dependency of dsDNA melting, a phenomenon counteracting and being counteracted by the influence of inorganic ions. Similar effects were reported from several other groups, for example, DNA helix destabilization by proline and its possible role in osmoadaptation [73], effects of phenoxazine derivatives [43] or lowering of dsDNA T_M _by trehalose [30]. Data of compatible solute influence on T_M _are presented in figure 2 and compared to those by sodium chloride and SYBR^®^-green, an intercalator. More complex structures like DNA triplices are stabilized by water structure forming solutes [74] but also by Hofmeister salts [62]. Even quadruplex/duplex equilibria are influenced by low molecular weight osmolytes [75,76]. While molecular crowding [77] is one important factor for quadruplex stability, the quadruplex/duplex transition is also induced by ions [61] and also effected by putrescin and polyethyleneglycol [78]. Finally, high molecular weight dextrans stabilize nonviral vectors during lyophilization at low osmolalities [79]. The above observations were obtained by the most simple scenario possible: a two component system with only solutes and nucleic acids. But to be able to understand how compatible solutes might act on nucleic acid stability, we need to clarify the fundamental principles first: The basics of nucleic acid stability and how this is influenced by physical parameters and other substances.

When thinking about nucleic acid stability the first thing which comes to mind is the DNA melting curve or, more precisely, *thermal *stability and *thermal *melting of dsDNA, which seems like a simple enough experiment. However, the simplicity behind this is deceiving. One is most likely tempted to forget about the influence of counterions, general ionic strength, solvent dielectricity and pH on dsDNA stability. In addition, other DNA structures and RNA are neglected completely. But even with a focus on thermal dsDNA melting the topic is quite complex [80-82] and, besides the melting point, we are able to gain thermodynamic data from a melting curve. Since even small PCR products can have complex melting profiles [83], the exact determination of melting temperatures might prove to be an arduous task with researchers being eager to find new efficient methods or to improve existing ones [71].

Thus, looking at the principles of nucleic acids stability does not really help us to find answers but points out even more factors which we have to keep in mind, as for example: the mechanics of base stacking and pairing [84], the main factors in DNA stability [85], intermediate states in DNA melting including bubble nucleation, cooperativity [86] and their fluctuation [87], local cooperativity in DNA melting [88] or sensitivity of T_M _to the presence of counterions [52]. All of these might or might not be affected by compatible solutes.

What other factors, besides compatible solutes, are known to influence nucleic acid stability? In 1999 Spink and co-workers demonstrated that the stability of DNA duplex and triplex structures not only depends on molecular forces such as base pairing or tripling or electrostatic interactions but also on its aqueous environment [89]. Thermal melting of RNA for example was shown to depend on pH and solvent [90]. Recent investigations point out the influence of mixed solvents and their different dielectric constants on DNA denaturation [91]. Such solvent-dependent effects indicate indirect effects on nucleic acid stability, possibly an influence of the solute or co-solvent on the structure of the solvent (water) or its electrochemical properties.

On the other hand, we are presented with reports on the proportionality of effects of intercalating substances on DNA duplex melting [42], which are quite similar to the linear dependency of solute concentrations and DNA melting points reported by Rees and co-workers [53]. Surfactants are known to interact with DNA intercalators and, depending on their surface charge, to intercalate or to affect T_M _to varying degrees [92]. SYBR^®^-green, commonly used in realtime PCR applications, even binds preferentially to certain DNA structures [93].

Again we have a huge amount of data and cannot really decide whether solutes have an indirect effect, interact directly with nucleic acids or both. To make the uncertainty complete, different compatible solutes might have different modes of action. But we are not completely left in the dark. Spink and co-workers have recently published calorimetric data and consider the importance of enthalpies of DNA melting in the presence of osmolytes [94].

## PCR improvement

In a polymerase chain reaction we have to take care of the proteins and their interactions with DNA in addition to thermal denaturation of the DNA template and formation of new DNA duplexes. To complicate matters the enzymatic reaction itself might be influenced by the addition of solutes or co-solvents, but we cannot ignore this aspect, especially since a lot of data concerning the influence of solutes are presently available. Additives which improve PCR reactions are highly sought after since even a simple "standard" application to amplify a certain region of DNA might not work properly with a particular sequence. Possible reasons might range from the simple, like GC-content or secondary structure formation of the template, to the obscure, like the complexity of compounds in a diagnostic PCR performed on clinical samples [95]. Cations, mainly K^+ ^and Na^+ ^are among the main inhibitors of a PCR and Mg^2+ ^– which is needed by the DNA polymerase – has a large, concentration dependent impact on PCR specificity [96].

It was the group of Weissensteiner which presented glycine betaine in 1996 as the first substance to counteract the effect of NaCl [27]. They introduced the term *cosolute *for such additives. Ever since betaine has been used as a PCR facilitator: as a single compound [29,97,98], in combination with DMSO [26,32,99-101] or together with BSA [102]. It has since been used on templates with varying GC content, to enhance formation of long PCR products [103,104] in low temperature PCR with heat labile polymerases [105] and in diagnostic PCR [106]. Sugars like trehalose or sucrose [107-109] have also been used with similar success as have been low molecular weight sulfones [110], amides [111] and sulfoxides [112]. In addition, recent data demonstrate that even synthetic derivatives of ectoines can act as powerful PCR enhancers [31].

Most of those reports lack a reasonable explanation as to how these compounds work. Some, for example sucrose and trehalose [108] or sarcosine [113], are supposed to stabilize the DNA polymerase. Betaine and again trehalose are believed to facilitate PCR by lowering T_M _or eliminating its dependency on base composition [30], which would be consistent with the early observation that DNA regions with high T_M _prevent amplification [114]. Furthermore, certain DNA sequences can cause DNA polymerase to pause, a phenomenon which is again counteracted by betaine [115]. More exotic substances like 7-deaza-2'-deoxyguanosine compete with dNTPs for the active site and slow down PCR [116]. This in turn gives a proofreading DNA polymerase more time to detect mismatched bases. But this is not to be confused with a true compatible solute, it is rather a PCR enhancer working on a different level.

Similar to the effects reported for nucleic acid stability and DNA melting we do not get a clear answer with respect to a possible mode of action. In spite of the large amount of reports on compatible solute effects in PCR we only get a vague picture as to what aspects of a PCR might be affected. And again we learn that different solutes react differently.

## Nucleic acid/protein-structures

Already in 1998 Record [117] included DNA protein interactions into his considerations on biophysical aspects of bacterial adaptation to osmolarity. With insufficient data to get a more detailed model, low water activity as the cause for molecular crowding and reduced biopolymer diffusion and interactions was proposed as the reason behind reduced growth rates. However, observing a whole cell might not be a good idea. Nucleic acid/protein structures without catalytic function would be more ideal targets for observations of compatible solutes effects. Unfortunately, this was only done in detail for the reconstitution of functional 50S ribosomes [34]. Though the model itself is not really simple and does not yield mechanistic information some impressive data were obtained: The authors demonstrated that, when using *in vitro *transcripts of *Escherichia coli *23S rRNA, ribosome reconstitution was stimulated by a factor of up to 100 in the presence of trimethyleaminoxide in combination with an antibiotic. More recent but less detailed data are available for a complex formed by leucine-responsive regulatory protein (LRP) with ribosomal DNA (rDNA) [118]. Here the CS ectoine stabilized the complex while the amino acid leucine had a destabilizing effect.

Returning to nucleic acid-protein interactions with catalytic activity I would like to draw your attention to restriction endonuclease complex formation and activity. Recent data show, that the dissociation of the *Eco*RI DNA complex is slowed down drastically by the neutral osmolytes glycine, glycerol, triethylene glycol and sucrose [119], an effect highly dependent on the resulting osmolarity and subsequent low water activity [120,121]. In another study glycine betaine was reported to improve restriction of DNA resistant to digestion despite the presence of appropriate recognition sites [39]. This was compared to the positive effect of betaine on PCR and contributed to the same – unknown – mechanisms. The opposite effect, protection to the point of complete inhibition of restriction, was observed for tetrahydropyrimidine derivatives and a range of type II restriction endonucleases [38]. The latter observation can be traced back to a patent application by the group of Lapidot [122,123]. In their work we find some NMR data which imply that proximity or binding of compatible solutes to guanine might play a role in this process. These findings are consistent with the general observation that CS have more pronounced effects on GC-rich sequences, which was made as early as 1993 [53]. Unfortunately the Lapidot group had published only one more project concerned with the influence of osmolytes on nucleic acid/protein interaction [124] and, apparently, did not continue NMR studies on the topic.

The findings of Lee and Gralla have a slightly more complex background: potassium glutamate takes part in the regulation of promoters for genes involved in osmoadaptation [125] and can even act as a global inhibitor of housekeeping genes under osmotic stress [126]. These contribute to changes in the DNA double helix structure, probably alteration of DNA bends, and depend on glutamate concentration. Related to this observation are data from the regulation of the betaine uptake system Bus in *Lactococcus lactis *[127]. The *in vitro *stability of a complex of regulator BusR and promotor *busA *strongly depended on the ionic strength of the buffer. Unfortunately no attempt was made to explain the molecular basis.

Naturally we would like to derive a general underlying concept for the influence of solutes on promoters. In this context it might be of interest to learn that the promoter region of a vast array of Human genes was reported to have a conserved set of distinct flexible and rigid regions independent of the consensus sequence [128]. Therefore, it would be challenging to test the influence of compatible solutes on mechanical properties of promoter regions, an aspect which so far has been neglected. A possible area worthy of investigation deals with solvent property changes, such as the effect of solvent dielectric constant on DNA torsional properties [129]. Again dielectric constants have not been reported for CS solutions.

## Models for molecular mechanisms

So far there is only one serious approach towards describing the molecular mechanisms of compatible solute action on nucleic acid properties, a fact which can be largely ascribed to the lack of basic mechanistic data. As already mentioned above, research on protein-solute interactions is more advanced and has led to a number of models. These might prove more or less useful for nucleic acids, depending on the general mode of interaction and the solute in question.

Prior to analysing those models against the background of nucleic acid research, we have to ask ourselves whether and up to which level we can treat nucleic acids and proteins alike. With a view to nucleic acid aptamers [130,131], the complexity of structures and surfaces of nucleic acids, in many ways similar to the antigen recognition site of the antibody F_ab _chain, seems to be comparable to that of proteins. Therefore, one might be tempted to assume similar macromolecular properties. But what about a comparison in detail?

In both cases we have a backbone/sidechain structure. However a phosphate/sugar backbone will behave differently in comparison to a polypeptide backbone, and only four hydrophobic bases offer less sequence flexibility than the 20 proteinogenic sidechains (derivatives of both species not counted). Similarly, it would be questionable to compare double-stranded helical DNA or RNA to alpha-helical or beta-strand like structures and other conformations to omega-loops, random coils and the like. Returning to the phosphates of the nucleic acids backbone, the reason for the most striking difference becomes apparent: nucleic acids always have a strong negative net charge, even when compared to acidic proteins, and thus are surrounded by an atmosphere of positively charged countercations.

In view of all those differences, what do nucleic acids and proteins have in common? The answer lies in the basic physical principles behind interactions, which include the surrounding water as solvent and which are for both biopolymers the driving force to fold. Therefore in general all models concerning those physical principles should be applicable to proteins and nucleic acids alike.

### Preferential interaction

The *preferential interaction theory *was phrased by Arakawa and Timasheff in 1983 [21] following the observation that both, glycerol and betaine, besides increasing the water surface tension are excluded from protein surfaces. The theory provides good explanations for a wide range of phenomena concerning the interactions of solutes, salts and biomolecules in water (though it might be better to say, *and *water). A revised version of this theory is given in reviews by Timasheff 1998 [132] and 2002 [133] with latest results from ion-exchange chromatography studies [134]. Recent publications also include molecular dynamics simulations showing ectoine preferential exclusion/interaction [135].

**Figure 3:**
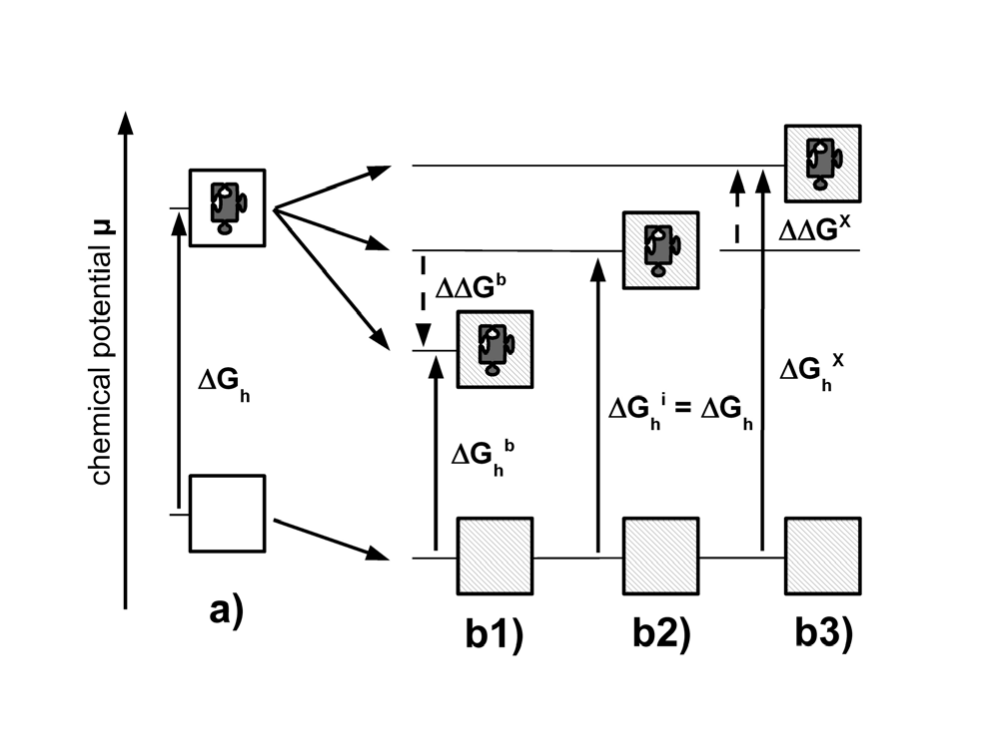
**Thermodynamics of preferential exclusion**. In water a) upon addition of a soluble protein the chemical potential μ is raised by the hydration energy ΔG_h_. A solution of solutes in water b) in general has a lower chemical potential. Depending on whether a solute preferredly binds b1) is inert b2) or is preferredly excluded hydration energies are lower (ΔG_h_^b^) equal (ΔG_h_^i^) or higher (ΔG_h_^x^) than ΔG_h_. Thus, in a theoretical experiment of transferring a protein from water into a solute solution we either gain energy ΔΔG^b^, have no energetic effect or have to put energy into the system ΔΔG^x^.

In short, if a protein is solubilised the chemical potential μ of the solution is raised by the free hydration energy ΔG_h_. Provided a solute of any kind is also present, we have a lower water activity and therefore a lower chemical potential. This effect is independent of the type of solute, but the number of particles (number of ions in salts) has to be considered. In the presence of solutes, as depicted in figure 3, there are three possibilities for the solubilization of the protein:

1) Solute molecules bind preferentially to the protein surface and, as a consequence, less water molecules bind. The protein is less hydrated and we observe a lower free hydration energy ΔG_h_^b ^< ΔG_h_. This is indeed a similar situation as in the *water replacement theory *[22] which will be presented next.

2) Solute molecules are inert and statistically distributed within the solution and on the protein surface. Free hydration energies ΔG_h_^i ^and ΔG_h _are the same.

3) The solute is preferentially excluded from the protein surface, equivalent to a state of preferential hydration. Total exclusion of the solute from the molecule surface would be one extreme and similar to a solution of protein in water. However this situation would only represent a similar but not an equal state, as we need to consider a thermodynamic difference in the surrounding medium, a concentration gradient of the solute from the bulk solution towards the protein. The free hydration energy ΔG_h_^x ^is higher than ΔG_h_.

Interestingly, nearly all known stabilizing solutes are excluded from the protein surface. The stability of a protein under denaturing influences is now determined by the difference ΔΔG = ΔG_h_^b, i or x ^– ΔG_h_. An inert solute will not have any influence at all, beside a general osmotic effect. A preferentially binding solute will destabilize the protein since ΔΔG is negative, and energy is released, which promotes unfolding of the protein. On the other hand, when solutes are excluded they have a positive ΔΔG, which means that unfolding needs additional energy and is inhibited.

### Water replacement

The *water replacement theory *by Clegg and co-workers [22] is based on the observation that many organisms are able to lose a larger amount of cellular water and return to full activity after rehydration. Cellular structures can be protected by the accumulation of certain compatible solutes and their interactions with surfaces. Since, in this model, water is replaced by solutes, it seems to be the complete opposite of the *preferential interaction theory*. However, we have to take into account that these observations were made with dried samples and therefore at very high solute concentrations. Replacement therefore, appears to be a very special situation of extreme low water activity, while the interaction model is valid for the more diluted range of solute concentrations. The importance of this model lies in the fact, that the relative affinity of a solute towards water or protein may well be concentration dependent, especially when desiccation (i.e. very low water activity) is involved [136].

### Water density fractions

The impact of macromolecules and compatible solutes on the three-dimensional structure of water hydrogen bond networks and *water density fractions *was first studied by Wiggins [23,137,138]. Effects like electrostatic attraction of anions by polycations (e.g. proteins) can prevent the equal distribution of a substance in water. Of course the same would be true for polyanions (nucleic acids) and cations. The resulting thermodynamic imbalance is then compensated by shifts in partial molar volume and therefore local density of water. This behaviour is unique for water and difficult to address experimentally since it is not measurable by classical physical methods.

In this theory low density water is supposed to have strong long range hydrogen bonds. They are weaker and more short-ranged in high density water. As a consequence the hydrophobic inner regions of a protein are better hydrated in high density water, *denaturation *is easier in the presence of *weaker *hydrogen bonds. If we now consider a solution of molecules which aggregate relatively large amounts of water in their hydration shell – like compatible solutes do – then this will create high density water fractions around the molecules and, subsequently, low density fractions elsewhere. Proteins in such an environment are now surrounded by low density water with *strong*, long range hydrogen bonds. This makes hydration of hydrophobic regions more difficult. Therefore, proteins are stabilized against unfolding.

The major drawback of this model is that the original idea behind it is based on structures of solid state water (ice) and experiments were done with gels. Therefore, we have to be very careful when trying to draw conclusions for the liquid state or even the situation *in vivo*. Nevertheless, though the model itself was not developed further, the concept is well worth considering [139,140] especially when dealing with surface effects [141].

### Osmophobic effect

Hydrogen bonds, van der Waals, electrostatic and hydrophobic interactions were long known to be responsible for protein folding when Liu and Bolen [24] discovered an additional force. The *osmophobic effect *becomes relevant in highly concentrated solutions – and in organisms which require high intraellular concentrations of osmolytes. In contrast to the hydrophobic effect which causes apolar amino acid residues to aggregate in the protein interior the *osmophobic effect *influences the conformation of the peptide backbone. The peptide backbone is preferentially excluded from a compatible solute solution, thus stabilizing the conformation of the backbone and preventing protein denaturation. This is in accordance with the *preferential interaction theory *[21] where the solutes are excluded from the protein surface. But in addition to the overall effect of destabilization/stabilization, with this model we are able to explore single thermodynamic effects. The molar transfer free energy of a substance ΔG^TR ^from water into a solute solution can be easily calculated as ΔG^TR ^= R × T × ln(c_W_/c_S_), with gas constant R, absolute temperature T and the ratio of maximum concentrations of the substance in water c_W _and in solute solution c_S_.

Using individual amino acids and diketopiperazine (cyclic di-glycine) as peptide backbone model, Bolen and co-workers observed that the major player was the osmophobic effect (on the backbone) with hydrophobic (side chain) for fine tuning. A current publication of the Bolen group discusses the prediction of energetics in folding and unfolding [142].

### Applying the protein models to nucleic acids

Current research on compatible solute protein interaction seems to converge from different points of view to a common concept. Both the Bolen and Record group postulate that the effects of osmolytes are based on the exclusion from or accumulation at polar peptide groups [142,143], with Street [143] breaking down the interactions into more simple physical properties and Courtenay [144] being the first to quantify those effects. Exclusion and accumulation are related to accessibility of the targets of those interactions. The solvent (and therefore solute) accessible surface area plays an important role. This brings us back to the point, that not only *preferential interaction *and *osmophobic effect *but also *water replacement *and maybe even *water density fractions *[141] are all related to surface effects. But how does this help us to apply the protein/compatible solute models to nucleic acids? As we seem to have a common basis in the protein models, we need to investigate a commonality shared by nucleic acids and proteins.

As a first approach, we could assume that surface features and the accessibility of those features is similar for proteins and nucleic acids. Simplifying things even more, we could treat nucleic acids or nucleic acid/protein complexes as structures with a uniform surface, as assumed for proteins in the *preferential interaction model*. Of course neglect of surface structure details is a weak point of this model and controversially discussed for proteins. Experimental data show that glycine betaine and urea, which are both denaturants of double stranded DNA, behave differently with respect to interaction [145,146]. While glycine betaine is preferredly excluded from the the negatively charged phosphate backbone [147], urea has to be regarded as an inert solute in a sense that its concentrations in the bulk and near the surface are the same. Different alcohols have been reported to be preferredly excluded from spermidine/DNA assemblies without affecting physical properties [148]. Especially with nonpolar alcohols we observe exclusion based on repulsive hydration interactions with the charged DNA surface and depending on the balance between alkyl carbons and hydroxyl oxygens [149].

Further experimental data can be collected from those special situations of low water activity where the *water replacement *model might be applied, in particular from freeze drying of DNA. Lyophilized cationic lipid-DNA complexes are reported to be stabilized best by disaccharides, while polysaccharides had no effect [33,150]. In a similar study with DNA loaded nanoparticles trehalose and glycerol were found to have the best effects among other sugars and polyols [151]. Stabilization was contributed to water replacement by the solutes. In contrast to the above, naked DNA was damaged during lyophylization, even in the presence of sugars. However, when a polycation was added, protection from degradation after spray freeze drying [152] was observed. Apparently, water replacement plays a role in DNA stability at low water activity but there must be other factors behind the observed protective effects.

Obviously both models have their use, but as we see, they also have their limitations. This is also true when we turn to *water density fractions*. Although three of the nucleic acids bases (adenine, thymine and guanine) have relatively low solubility in water they are not really hydrophobic, we know that all bases form hydrogen bonds. Therefore, we would expect dissociation of double strands and similar structures to be easier in low density water. And indeed, as discussed above, a range of solutes which stabilize proteins against unfolding lower the melting point of DNA (e.g. [30,43,53,73,74]). But the model cannot be the sole explanation for osmolyte effects on nucleic acid/protein-complexes. Especially stabilization of such complexes [34] has to include additional aspects because solutes which stabilize proteins *de*stabilize the nucleic acid, at least they do destabilize double helical structures in the sense that they lower T_M_. As demonstrated, lowering of the water activity by solutes might inhibit dissociation and override the destabilization [145,146].

This leaves us with the *osmophobic effect*. Here the interesting question is, whether we have osmophobic effects at all with nucleic acids. Due to its extremely high solubility in water one would not suspect the highly polar phosphate/sugar polyanion to qualifiy as an osmophobic element. But the term *osmophobic *as used in the model rather relates to the behaviour of the (macro)molecule in the presence of cosolvents than to general solubility. In continuation of their research on ion/RNA interactions [50] the Draper group very recently investigated the influence of compatible solutes on a diversity of RNA structures [25]. Using the concepts of Bolen and Record thermodynamics could be resolved sufficiently. Stabilization of RNA tertiary structure by glycine betaine was shown to correspond with complete exclusion of betaine from the backbone, which is exactly the same situation as for proteins. With such an effect we can also easily explain destabilization of dsDNA (resulting in a lower T_M_) if we postulate that corresponding ssDNA structures are more stable than the duplex in presence of a solute. But is this the whole story? It is admitted in the survey by Lambert that the model is "simplifying" the situation, especially with respect to counterions. And although all fits nicely for betaine we have already encountered dsDNA denaturants like urea that behave differently with respect to interaction with the backbone. As already shown more than 30 years ago [153,154] in the presence of certain compounds nucleic acid bases tend to be more easily exposed in an environment, and we can draw the conclusion that protein stabilizing solutes which destabilize DNA may have a similar effect. So in addition to a possible osmophobic element, the nucleic acid backbone, we can characterize the bases as an ***osmophilic ***element.

### More questions

If we want to go beyond calculations and understand the mechanistics behind the complex interactions, even Drapers' concept, valuable as it is, leaves questions unanswered. Especially when it comes to the point of the counter ion atmosphere we are at the most striking difference between nucleic acids and proteins, which shows us that the surfaces of proteins and nucleic acids are in fact *not *very similar. We can draw on a wealth of information about ions in general, and also about the role of counterions or ionic strength in nucleic acid or nucleic acid/protein complex properties [25,36,50,52,59-62,127]. The phosphate backbone or, to be more precise, its strong negative charge and the atmosphere connected to it, are very likely both interacting partners for (compatible) solutes. So we cannot leave this aspect unattended.

In this context we have to pay special attention to anioic organic solutes which are accumulated in particular by thermophilic and hyperthermophilic microrganisms [155], potassium presumably serving as counterion. Unfortunately we do know even less about the effects anionic solutes have on nucleic acids than we know about the effects of zwitterionic and uncharged species; nevertheless, positive effects in nucleic acid applications have been reported [4,156].

Since nucleic acid structures are already destabilized by the elevated temperatures of (hyper-)thermophilic habitats an additional destabilizing effect would probably be fatal *in vivo*. The interesting question is, do anionic solutes have special properties to be able to stabilize both proteins and nucleic acids against thermal denaturation? Obviously, due to the negative charge of the phosphate backbone electrostatic repulsion makes direct interactions between nucleic acids and anionic solutes quite improbable. On the other hand, osmophilic or osmophobic effects do not necessarily depend on direct interactions but describe the general behaviour of a (macro)molecule in the presence of a (compatible) solute. And we should not forget about the potassium cations which might have to work *in concert *with the organic anion for the best effect in protein and DNA stabilization.

Things get even more exiting when looking at organisms dealing with thermal and osmotic stress since they have to balance adaptation to both conditions. We know that some of the organic anions like glycosylglycerate do have osmoprotective properties while others like mannosylglycerate are solely for thermoprotection [155] with trehalose serving as the main osmoprotectant [157]. But a lack of experimental data does not allow for more than speculation.

A third point is that all the models presented above deal with possible indirect effects of CS on biopolymers, but at least tetrahydropyrimidines have also been reported to interact more directly with DNA [122,123]. Interestingly, these compounds seem to prefer interactions with guanine, a property which perfectly correlates with their increased impact on GC-rich sequences. Thus, indirect interactions are not the whole story either. At a first glance, the structural similarity of the tetrahydropyrimidines with the pyrimidine bases may indicate the ability to intercalate into the base stacks. One should however be aware of the fact that these solutes are not completely planar but rather resemble a half-chair conformation. Trying to understand potential direct interactions of tetrahydropyrimidines with other biomolecules we can obtain useful information from protein biochemistry. The recent crystallization and X-ray analysis of substrate binding proteins of osmolyte transporters revealed a pocket of aromatic residues as the binding motif. In ProX [158] this pocket is formed by four tyrosines and in OpuAC by three tryptophanes [159]. The fact that binding of glycine betaine and proline betaine is apparently established by cation-π-interactions and non-classical hydrogen bonds provides us with a means to anticipate direct interactions with the nucleic acid bases.

In addition we cannot fail to notice an influence of overall bulk dielectric constant ε' on DNA properties [129]. And in this context it is known for a long time how drastic ε' can be changed by small organic compounds [160,161].

## Mathematical and Computer methods

So far there are neither publications proposing a general concept for thermodynamic calculations of compatible solute effects on nucleic acids nor molecular dynamics simulations thereof. A recent publication by Rösgen [162] discusses the basics of solute protein and solute protein metabolite thermodynamics and might prove to be a good starting point for similar considerations towards solute nucleic acid interactions. Closest to calculations concerning nucleic acids and solutes come a thermodynamic model including the effects of salt concentration on nucleic acid duplex-simplex transitions [163] and molecular dynamics studies of ion distributions for DNA duplexes and clusters [164]. But as discussed above, the influence of solutes also arises from indirect effects, mainly as a consequence of a modulation of the properties of the solvent water. The thermodynamics of force-induced melting of DNA double helices, for example, including indirect effects of solvents and solutes [165,166] are well known. In addition the thermodynamics of tRNA microhairpin stability and its solvent dependence have been investigated [90,167]. Some earlier publications already discussed solvent effects on nucleic acid base associations and simulations thereof [168,169] as well as general DNA dynamics in aqueous solutions [81]. Modes of direct interaction of compatible solutes which display structural similarities to DNA, like the tetrahydropyrimidines, might be derived from considerations on DNA aggregates and their melting behaviour [170]. More general models for DNA melting, which consider base pairing and stacking [84], bubble nucleation and cooperativity [86,88] or bubble relaxation [87] and inhomogeneity of DNA melting [171] are permanently under improvement. And a closer investigation into the secondary structure of nucleic acids [172] and their involvement in nucleic acid/protein interactions [173] might help us to understand how compatible solutes affect nucleic acid/protein complexes and their formation.

## Conclusion

Compatible solute effects on nucleic acid properties and nucleic acid/protein complexes have been known for some time now. Besides their exploitation for *in vitro *applications they are also of great significance *in vivo *and have a large impact on vital functions. In contrast to the large amount of application data, we know only little about the molecular background of the observed effects. What is missing is a broad systematic approach to basic data, especially such simple things like thermal melting and stability. Nevertheless, we do know more than one might suspect from a quick glance at present day research.

One major problem is the comparability of data, which even starts at simple thermal melting experiments. In a system where buffer composition strongly affects the properties of the subject under investigation we have to expect three- (or more-) way interactions between target (nucleic acid), buffer and effector (compatible solute). Therefore, a broader coherent set of basic data is definitely needed before we can start to interpret the results of more sophisticated experiments in a proper way, which is especially true for anionic organic solutes.

**Figure 4:**
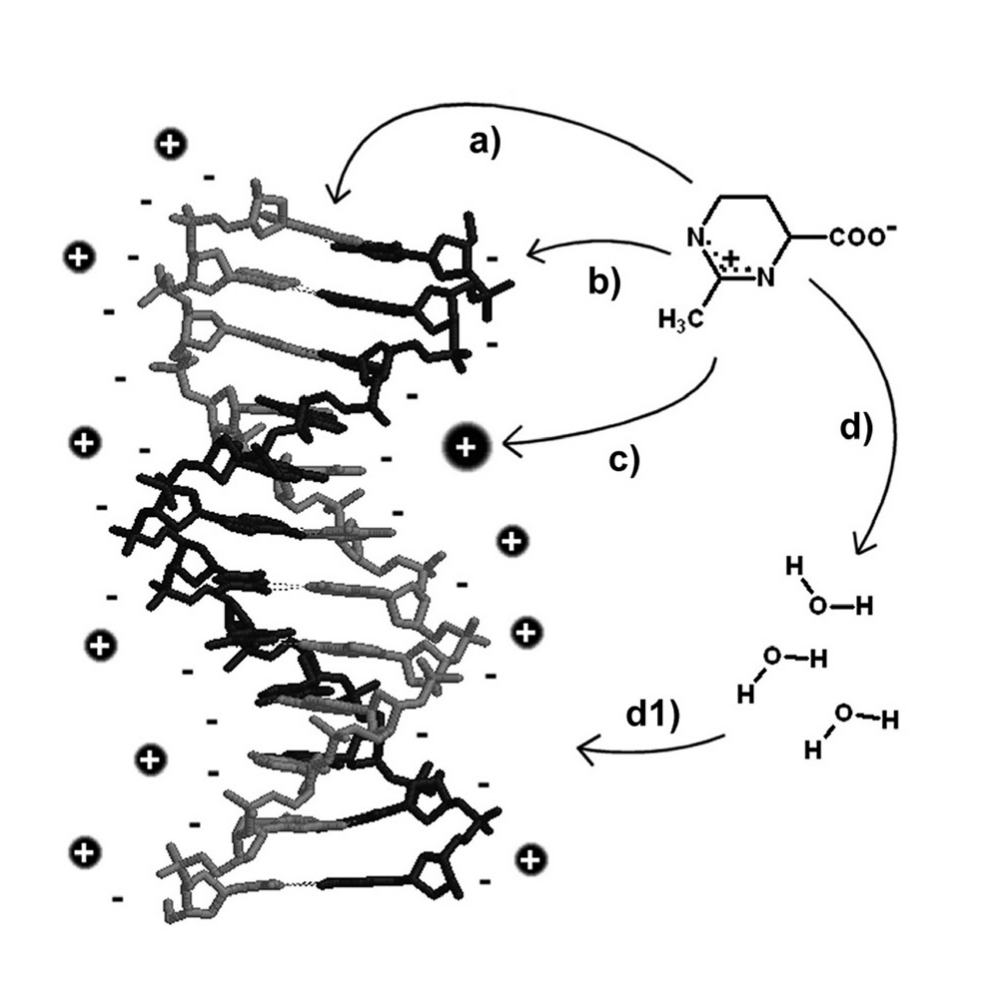
**Interactions of compatible solutes with DNA**. Interactions can be direct with a) the single bases, base pairs and stacks and b) the negatively charged phosphate-sugar-backbone or indirect by influence on c) the counterions and d) the solvent properties which in turn then influence the DNA.

Building a model to describe interactions between compatible solutes and nucleic acids is a difficult task, because the topic is far more complex than interactions between solutes and proteins. As summed up in figure 4, we possibly have to deal with a multitude of effects:

a) direct interactions with nucleic acid bases, probably due to cation-π-interactions similar to the situation in the solute binding proteins

b) direct interactions with the negatively charged phosphate backbone

c) interactions with the positively charged counterions and

d) indirect interactions via changes in solvent properties.

When investigating interactions with nucleic acid/protein-structures things get even more complex, since we have to consider not only the effects on nucleic acids, but also interactions of solutes with the protein(s) and the influence of osmolytes on the interactions between nucleic acids and proteins. *In vivo *of course, all other metabolites add to the whole picture.

We can use the concept that the effects of osmolytes are based on the exclusion or accumulation at surface elements for calculation of thermodynamics as demonstrated by Lambert [25], and all four of the models presented here have their – however limited – use for compatible solute/nucleic acid interactions. But – since those models are developed for protein/solute interactions – direct interactions are to a large extent excluded. Another point left out is the negative charge of the phosphate backbone and its counterion atmosphere. And lastly, in analogy to the osmophobic effect of proteins we might want to mint a new term here: The potential **osmophilic effect **of nucleic acid bases.
